# Chromosome-level genome assembly of the stonefly *Rhopalopsole triangulispina* Mo and Li, 2025 (Plecoptera: Leuctridae)

**DOI:** 10.1038/s41597-026-06631-7

**Published:** 2026-01-23

**Authors:** Aili Lin, Jinjun Cao, Dávid Murányi, Ding Yang, Weihai Li, Raorao Mo

**Affiliations:** 1 Henan International Joint Laboratory of Taxonomy and Systematic Evolution of Insecta, Henan Institute of Science and Technology, Xinxiang, Henan 453003 China; 2 Observation and Research Station on Water Ecosystem in Danjiangkou Reservoir of Henan Province, Nanyang, Henan 474450 China; 3 Department of Zoology, Eszterházy Károly Catholic University, Leányka u. 6, Eger, H-3300 Hungary; 4 Department of Entomology, China Agricultural University, 2 Yuanmingyuan West Road, Beijing, 100193 China

**Keywords:** Entomology, Sequence annotation

## Abstract

The superfamily Nemouroidea (Plecoptera) represents one of the most diverse and ecologically significant groups of stoneflies, with nymphs serving as crucial bioindicators of freshwater ecosystem health due to their sensitivity to water quality. However, the evolutionary and genomic studies of this group have been hindered by the lack of high-quality reference genomes. Here, we present a chromosome-level genome assembly for *Rhopalopsole triangulispina* Mo and Li, 2025 within Nemouroidea, generated by integrating PacBio HiFi long reads, Illumina short reads, and Hi-C chromatin interaction data. The final assembly spans 347.119 Mb with a scaffold N50 of 27.479 Mb, and 96.91% (336.39 Mb) of the genome is anchored to 13 pseudochromosomes. BUSCO assessment reveals a high completeness of 98.4% (insecta_odb10). The genome contains 48.50% repetitive elements (168.35 Mb) and encodes 12,857 protein-coding genes, which were comprehensively annotated using homology, transcriptomic, and ab initio evidence. This high-quality genome provides a foundational resource for resolving phylogenetic relationships within Nemouroidea, advancing studies on insect genome evolution, and enhancing freshwater biomonitoring efforts through genomic tools.

## Background & Summary

Nemouroidea is one of the most abundant and diverse superfamilies within the order Plecoptera, comprising over 1,500 known species distributed worldwide, except in Antarctica^1,2^. It exhibits hemimetabolous development, with nymphs that are predominantly aquatic, inhabiting benthic habitats in clean, fast-flowing streams, lakes, and ponds—often under stones or in muddy sediments. Adults have weak flight capabilities and typically remain near water, occurring on riparian rocks, vegetation, and structures such as tree branches and stone bridges^3^. Nymph growth, development, reproduction, and distribution are strongly influenced by environmental factors including water temperature, dissolved oxygen levels, chemical composition, clarity, substrate type, aquatic biota, and surrounding vegetation. Owing to their sensitivity to water quality, Nemouroidea nymphs are key bioindicators for monitoring and assessing freshwater ecosystem health^4–7^.

One notable family within Nemouroidea is Leuctridae, which includes approximately 13 genera and 400 species recorded worldwide^1^. Leuctridae is primarily distributed in mountainous regions across the Palearctic, Nearctic, and Oriental biogeographic realms. A significant genus within this family is *Rhopalopsole* Klapálek, 1912^8^, which occurs in the Oriental and eastern Palearctic regions and accounts for about one-quarter of the family’s species diversity^1^. The nymphs mostly inhabit clear, well-aerated streams and mountain brooks. Due to their sensitivity to water quality changes, these nymphs serve as important bioindicator taxa in freshwater biomonitoring. China is the country with the highest species diversity of *Rhopalopsole*, with over 60 known species^3,9^. Currently, 14 whole genomes of species within the mayfly superfamily Nemouroidea have been published in the NCBI database, including 6 at the chromosome level, 3 at the scaffold level, and 5 at the contig level. However, none of these genomes have been annotated. The lack of genome annotation limits their application in areas such as gene functional studies, comparative genomics, and evolutionary research. In addition, the phylogenetic relationships within the superfamily Nemouroidea remain unclear, and previous studies have primarily relied on morphological characters^10–12^, mitochondrial genomes^13,14^, and transcriptomic data^15,16^.

In this study, we integrated PacBio HiFi, Illumina, and Hi-C sequencing technologies to assemble the chromosome-level genome of *Rhopalopsole triangulispina* Mo and Li, 2025 for this superfamily, and comprehensively annotated its repetitive sequences, non-coding RNAs, and protein-coding genes. The availability of this high-quality genome not only provides crucial data for resolving the phylogenetic relationships within Nemouroidea but also lays an important foundation for studying genome architecture and evolution in stoneflies (Plecoptera).

## Methods

### Sample collection and sequencing

Adult specimens of *Rhopalopsole triangulispina* were collected using a sweep net on November 26, 2024, at the Shagang Station of the Qianjiadong National Nature Reserve in Guanyang County, Guilin City, Guangxi, China (elevation: 408 m; 25°25′28″N, 111°12′15″E). After sex identification, the specimens were surface-cleaned and immediately flash-frozen in liquid nitrogen for subsequent omics analyses. Genomic DNA was extracted from the head (excluding the abdomen) of a female specimen using a modified CTAB method. DNA quality was comprehensively assessed using NanoDrop spectrophotometer (Thermo Fisher Scientific, ND-2000), Qubit fluorometer (Invitrogen, Q33238), and pulsed-field gel electrophoresis (Biorad, 1703672) to ensure sufficient integrity and purity for library construction.

For Illumina short-read sequencing, a paired-end library with an insert size of approximately 350 bp was constructed using the VAHTS Universal Plus DNA Library Prep Kit, Sand sequencing was performed on an Illumina Xplus platform with a sequencing coverage of approximately 80X. Library preparation was carried out by Berry Genomics Corporation (Beijing, China).

For PacBio HiFi long-read sequencing, the library was constructed using the SMRTbell^®^ Express Template Prep Kit 3.0 (Pacific Biosciences, #PN 101-853-100, CA, USA). Genomic DNA was first sheared to an average size of ~20 kb using a Megaruptor instrument (Diagenode, B06010001, Liege, Belgium), followed by size selection and concentration using AMPure^®^ PB Beads (Pacific Biosciences, 100-265-900). The library preparation included end-repair, damage repair, A-tailing, hairpin adapter ligation, and exonuclease digestion. Size selection was performed using the SageELF system (Sage Science, ELF000). The HiFi library was prepared by Berry Genomics Corporation (Beijing, China) and sequenced on a PacBio Sequel Revio platform with a sequencing coverage of approximately 40X.

For transcriptome sequencing, full-length RNA was extracted using the DP441 RNA prep Pure Plant Plus Kit. Oxford Nanopore Technologies (ONT) libraries were constructed using the SQK-PCS109 and SQK-PBK004 kits, followed by sequencing on the Oxford Nanopore PromethION platform. Library preparation was performed by BenaGen (Wuhan, China). For Illumina short-read RNA sequencing, total RNA was extracted using TRIzol™ Reagent. A stranded mRNA-seq library was constructed using the VAHTS mRNA-seq v2 Library Prep Kit, and sequencing was carried out on the Illumina Xplus platform. Library preparation was conducted by Berry Genomics Corporation (Beijing, China).

Additionally, the Hi-C library was constructed by Berry Genomics Corporation (Beijing, China), involving steps including formaldehyde cross-linking, digestion with the MboI restriction enzyme, end repair, ligation for circularization, and DNA purification. The Hi-C library was sequenced on the Illumina Xplus platform. An overview of sequencing data volume and coverage depth for the *R. triangulispina* genome project is provided in Table 1.

**Table 1:** Statistics of the sequencing data used for genome assembly.

Library type	Insert sizes (bp)	clean data (Gb)	Sequencing Platform	Sequencing coverage (x)	SRA Accession Number
WGS	350	26.21	Illumina Xplus	81.07	SRR35229752
PacBio HiFi	~20 kb	12.76	PacBio Sequel Revio	39.48	SRR35229750
Hi-C	300–800	39.18	Illumina Xplus	121.21	SRR35229751
RNA-sr	350	22.18	Illumina Xplus	—	SRR35229748
RNA-ONT	—	11.12	Oxford Nanopore PromethION	—	SRR35229749

### Genome assembly

**Fig. 1:**
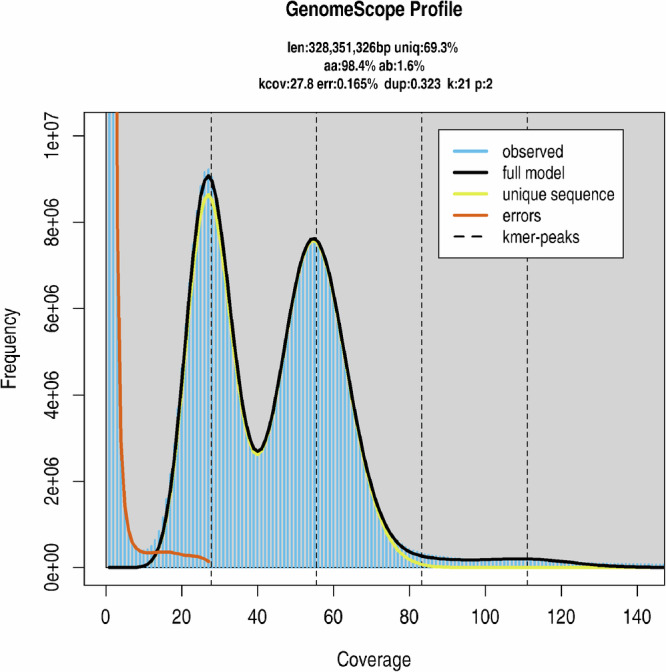
GenomeScope genome size estimates for *Rhopalopsole triangulispina*.

Genome survey analysis was performed to estimate genome size, heterozygosity, and repeat content, providing critical parameters for downstream assembly strategies. Illumina short reads were first quality-controlled using fastp v0.23.4^17^ (parameters: -q 20 -D -g -x -u 10 -5 -r -c) to retain bases with Q20 or higher quality, remove low-quality bases, adapter sequences, and poly-G/X tails, and correct bases using overlapping regions. K-mer analysis (k = 21) was performed, and the genome size and heterozygosity of R. triangulispina were estimated using GenomeScope v2.0^18^. The analysis was run with a maximum k-mer coverage of 10,000 (command: Rscript genomescope.R -i khist.txt -o out10000 -k 21 -p 2 -m 10000), yielding an estimated genome size of 328.35 Mb and a heterozygosity rate of 1.61% (Fig. 1).

High-quality HiFi reads (retaining sequences with Q20 or higher base quality) were generated using pbccs v6.4.0 and assembled with Hifiasm v0.24.0^18^ (parameters: hifiasm -o hifi -t 32–dual-scaf ccs.fa). To eliminate potential contamination or erroneous sequences, only contigs with sequencing depth ≥4X (i.e., excluding fragments below 1/10 of the average depth) were retained. To address potential heterozygous duplications, Purge_dups v1.2.5^19^ was applied with default parameters (−2 -a 70) to remove redundant sequences based on sequence similarity and depth. Minimap2 v2.29^20,21^ was used for sequence alignment, with parameters -x map-hifi for read mapping and -x asm5 -DP for self-alignment of the assembly.

**Fig. 2:**
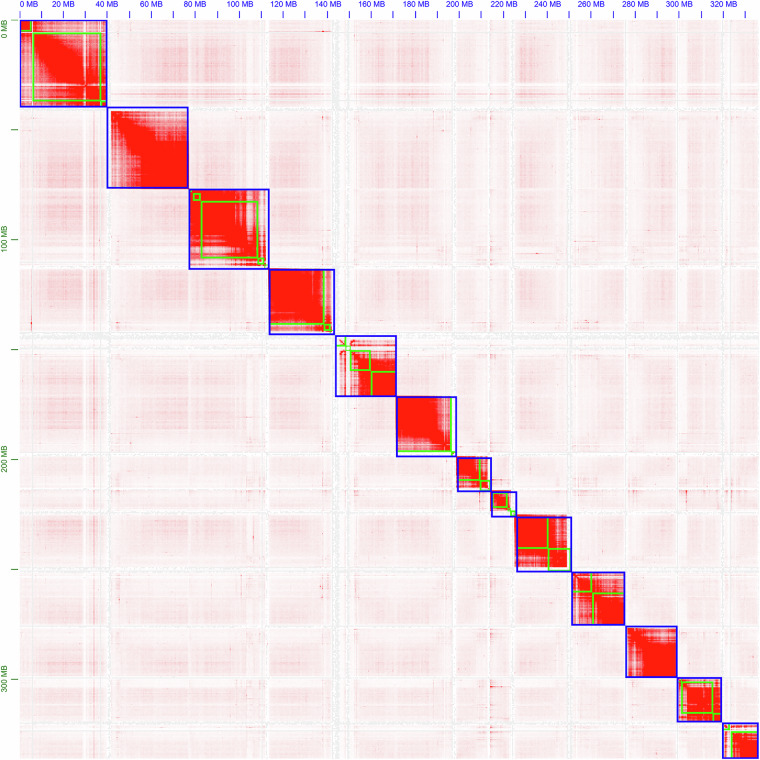
Genome-wide chromosomal heatmap of *Rhopalopsole triangulispina*.

Chromosome-level scaffolding was achieved using Hi-C data through the YAHS v1.2^22^ pipeline (parameters: ~/tools/yahs-1.2/yahs -e GATC contigs.fa aln.bam; ~/tools/yahs-1.2/juicer pre -a -o out_JBAT yahs.out.bin yahs.out_scaffolds_final.agp contigs.fa.fai). Hi-C reads were first aligned, deduplicated, and interaction pairs extracted using chromap v0.2.6^23^ (parameters: chromap–preset hic -x contigs.index -r contigs.fa -1 “*.R1.fastq.gz” -2 “*.R2.fastq.gz”–SAM -t 32–remove-pcr-duplicates -o aln.sam). Two rounds of scaffolding were performed. The initial scaffolding results were manually inspected and corrected for misjoins, inversions, and translocations using Juicebox v1.11.08^24^ (parameters: java -jar juicer_tools.jar pre out_JBAT.txt out_JBAT.hic chrom.sizes) before final scaffolding. Sequencing depth for each pseudochromosome was calculated using SAMtools v1.10^25^ (parameters: samtools depth -r chr chr.bam) based on alignments of either HiFi or Illumina WGS reads. The Hi-C interaction heatmap (Fig. 2) demonstrates high-quality scaffolding, resulting in 13 chromosome-level scaffolds.

The final *R. triangulispina* assembly spans 347.119 Mb (close to the survey estimate), with 88 contigs and 42 scaffolds, contig and scaffold N50 values of 15.378 Mb and 27.479 Mb, respectively, and a maximum scaffold length of 40.341 Mb. The GC content is 39.10% (Table 2). The 13 pseudochromosomes collectively span 336.39 Mb, achieving a scaffold anchoring rate of 96.91% (Table 3). Genome completeness was assessed using BUSCO v5.7.1^26^ with the insecta_odb10 database (n = 1,367 single-copy orthologs), yielding a completeness of 98.4% and a duplication rate of only 1.0%, indicating a highly complete assembly with low redundancy. The mapping rates of Illumina genomic reads, transcriptomic reads, and PacBio RNA and HiFi reads all exceeded 95% (95.46%, 96.07%, 99.59%, and 99.46%, respectively), demonstrating high assembly reliability (Table 2). Potential contamination was screened using MMseq. 2 v13^27^ by aligning against the NCBI nt and UniVec databases. Single-base accuracy (QV value) and k-mer consistency were evaluated using Merqury v1.3^28^, with QV values for all chromosomes exceeding 60 (error rate <1 × 10^−7^) (Table 3). Collectively, these metrics indicate that the *R. triangulispina* genome assembly meets the standards for high-quality, chromosome-level assemblies in terms of continuity, completeness, and accuracy.

**Table 2:** Genome assembly statistics for *Rhopalopsole triangulispina*. S: single BUSCOs; D: duplicated BUSCOs; F: fragmented BUSCOs; M: missing BUSCOs.

Content	Values
**Genome Assembly**	
Assembly size (bp)	347,118,714
Number of pseudo-chromosomes (sizes)	13 (336,391,789 bp)
Number of scaffolds/contigs	42/88
Longest scaffold/contig (Mb)	40.341/37.155
N50 scaffold/contig length (Mb)	27.479/15.378
GC content (%)	36.39
**BUSCO completeness (%)**	98.4
S	97.4
D	1.0
F	0.2
M	1.4
**Mapping ratio of reads (%)**	
Illumina WGS	95.46
HIFI	99.46
RNA-sr	96.07
RNA-ONT	99.59

**Table 3:** Genome assembly statistics of length, sequencing coverage and QV value for each chromosome of *Rhopalopsole triangulispina*.

Chromosome	Length (bp)	HiFi (X)	WGS (X)	QV
Chr01	40340739	39.4318	78.5329	68.5511
Chr02	37154854	38.0366	76.9548	71.5857
Chr03	36381375	38.5289	83.4962	68.2259
Chr04	30016908	38.0373	78.7149	68.9878
Chr05	28011445	31.8983	66.1743	69.2674
Chr06	27478658	36.1826	73.8038	69.6187
Chr07	25193401	36.3458	76.3507	65.5946
Chr08	24728049	35.101	69.9718	69.0851
Chr09	23359996	36.7106	76.1722	68.4105
Chr10	20368668	36.6843	77.4344	65.877
Chr11	16710534	33.2182	63.0079	68.0276
Chr12	15287389	34.7126	68.538	75.0254
Chr13	11359773	36.9445	68.2377	71.0363

### Genome annotation

To construct a species-specific repeat library for *R. triangulispina*, RepeatModeler v2.0.5^29^ was employed with the LTR structural identification module enabled (-LTRStruct), combining structural features and *de novo* prediction to identify repetitive elements. This custom library was merged with the Dfam 3.8^30^ and RepBase-20181026^31^ databases to generate a comprehensive repeat reference database. Subsequently, RepeatMasker v4.1.5^32^ (parameters: RepeatMasker -e ncbi -pa 11 -xsmall -lib repeatlib.fa genome.fa) was used in conjunction with this database to annotate repetitive sequences across the genome. To further investigate the evolutionary dynamics of transposable elements (TEs), the Kimura 2-Parameter divergence for each TE family was calculated using the RepeatMasker-associated script calcDivergenceFromAlign.pl, providing insights into their expansion history. The results revealed a total of 772,818 repetitive elements in the *R. triangulispina* genome, spanning 168,350,053 bp and accounting for 48.50% of the assembly, classifying it as a genome with typical medium-to-high repeat content. The major repeat types identified include: unknown elements (20.92%), DNA transposons (8.37%), SINEs (5.56%), LTR retrotransposons (3.59%), and simple repeats (3.63%) (Table S1).

Non-coding RNA (ncRNA) annotation was performed using two complementary approaches: (1) Homology-based identification: Conserved ncRNAs, including rRNAs, snRNAs, and miRNAs, were identified and annotated using Infernal v1.1.5^33^ against the Rfam database. (2) tRNA prediction: tRNAscan-SE v2.0.12^34^ was used to comprehensively predict tRNAs in the genome, with low-confidence predictions filtered out using the built-in script ‘EukHighConfidenceFilter’ to ensure annotation accuracy. In total, 2,411 non-coding RNA genes were annotated in the *R. triangulispina* genome, comprising 1,153 rRNAs, 60 miRNAs, 69 snRNAs, 297 tRNAs, 1 ribozyme, and 2 lncRNAs. Among them, the snRNAs include 38 spliceosomal RNAs (U1, U2, U4, U5, U6, U11), 2 minor spliceosomal RNAs (U4atac, U6atac), 21 C/D box snoRNAs, and 4 H/ACA box snoRNAs (Table S2).

Protein-coding gene structure prediction was performed using the MAKER v3.01.04^35^ pipeline, integrating three lines of evidence for comprehensive analysis: (1) Ab initio prediction: BRAKER v3.0.6^36^ and GeMoMa v1.9^37^ were used for independent predictions, and their results were merged into a single input file for MAKER to expand the candidate gene set. BRAKER integrated transcriptomic data (generated by aligning Illumina RNA-seq reads using minimap2 with the -x splice:sr parameter to produce BAM files) and arthropod protein sequences (from OrthoDB11 database^38^) to automatically train Augustus v3.4.0^39^ and GeneMark-ETP^40^, thereby improving prediction accuracy. GeMoMa predictions were based on protein homology and conservation of intron positions. Multiple closely related species were selected as references, including the paleopteran Ischnura elegans, the holometabolous *Drosophila melanogaster* (Diptera), the hemimetabolous *Rhopalosiphum maidis*, and three polyneopteran insects: *Anabrus simplex*, *Bacillus rossius redtenbacheri*, and *Periplaneta americana*. Parameters were set as GeMoMa.c = 0.4 and GeMoMa.m = 67000. (2) Transcriptome-supported gene prediction: StringTie v2.2.1^41^ was used in “mixed assembly” mode (–mix) to perform reference-guided assembly of both Illumina and ONT long-read RNA-seq data. The Illumina RNA-seq BAM file was derived from the minimap2 alignment described above, while the ONT RNA-seq data were aligned using minimap2 (-x splice) to generate the input BAM file. (3) Homology-based prediction: High-quality protein sequences from the aforementioned related species were used in homology searches to assist in gene model construction, enhancing the identification of conserved coding regions.

Integrating all three lines of evidence, MAKER predicted a total of 12,857 protein-coding genes in the *R. triangulispina* genome. The average gene length is 13,072.4 bp, containing an average of 7.9 exons (mean length 315.8 bp) and 6.9 introns (mean length 1,612.3 bp), with each gene harboring an average of 7.6 CDS segments (mean length 224.4 bp) (Table 4). BUSCO assessment of the predicted protein sequences yielded a completeness score 98.4% [single buscos: 78.6%, duplicated buscos: 19.8%], with only 1.5% missing, consistent with the genome-level BUSCO results, indicating high completeness in gene structure annotation.

**Table 4:** The results of protein-coding gene annotation for *Rhopalopsole triangulispina*.

annotation	Number
**Structure annotation**	
Number of protein-coding genes	12,857
Number of predicted protein sequences	17,186
Mean protein length (aa)	597.4
Mean gene length (bp)	13,072.4
Gene ratio	48.42%
Number of exons per gene	7.9
Mean exon length (bp)	315.8
Exon ratio	9.33%
Number of CDSs per gene	7.6
Mean CDS length (bp)	224.4
CDS ratio	6.34%
Number of introns per gene	6.9
Mean intron length (bp)	1,612.3
Intron ratio	39.09%
**Function annotation**	
Number of genes matching Uniprot records	11,804
Number of genes labelled as “Uncharacterized protein”	696
Number of genes labelled as “unknown function”	1,106
Number of genes with InterProScan annotations	10,647
Number of genes with GO items from InterProScan annotations	6,440
Number of genes with eggNOG annotations	11765
Number of genes with GO items from eggNOG annotations	8882
Number of genes with Enzyme Codes (EC) from eggNOG annotations	2,765
Number of genes with KEGG ko terms from eggNOG annotations	7,911
Number of genes with KEGG pathway terms from eggNOG annotations	4,847
Number of genes with COG Functional Categories from eggNOG annotations	11,099
Number of genes with GO items (combining InterProScan and eggNOG results)	9,989
Number of genes with KEGG pathways items (combining InterProScan and eggNOG results)	4,847

**Fig. 3:**
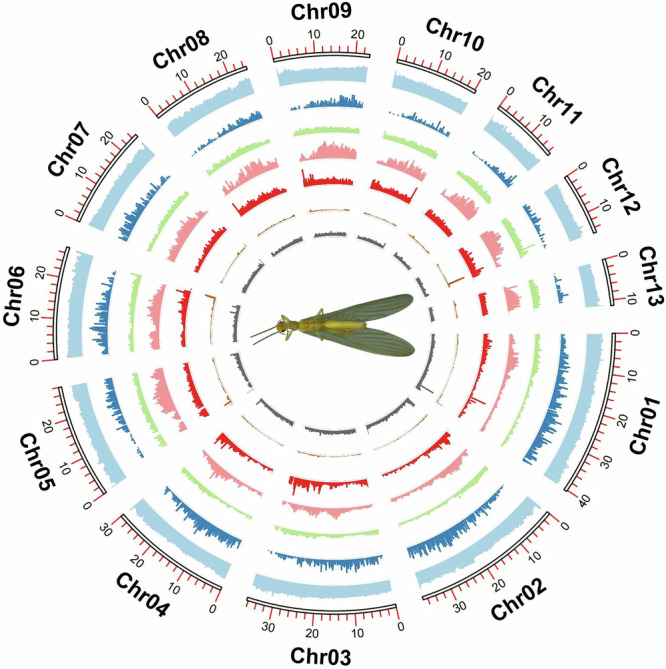
Circos plot showing the genomic characters of *Rhopalopsole triangulispina* from outer to inner: chromosome length, GC content, gene density, DNA transposon density, SINE density, LINE density, LTR density, and simple repeat density, famale adult of *Rhopalopsole triangulispina*.

Functional annotation of the predicted genes was performed using the following strategies: (1) Sequence homology search: Diamond v2.1.7.161^42^ was used in high-sensitivity mode (–very-sensitive -e 1e-5) to align against the UniProtKB (Swiss-Prot + TrEMBL) database to assign functional descriptions. A total of 11,804 genes (91.80%) received significant hits. (2) Domain and pathway analysis: InterPro v5.70-102.0^43^ was employed to search Pfam^44^ and other databases for conserved protein domains, successfully annotating 10,647 genes. eggNOG-mapper v2.1.12^45^ was used to query the eggNOG v5.0.2^46^ database for Gene Ontology (GO) and KEGG/Reactome pathway annotations, assigning GO terms to 9,989 genes and KEGG pathways to 4,847 genes. All functional annotation results were integrated into a unified GFF file. Genome feature visualization was performed using TBtools-II v2.096^47^ to generate a circular plot (Fig. 3), depicting from outer to inner rings: chromosome length, GC content, gene density, and the distribution densities of various repeat types (DNA transposons, SINEs, LINEs, LTRs, and simple repeats), providing a comprehensive view of the genome’s structural characteristics.

## Data Records

The *R. triangulispina* genome project has been deposited in the NCBI database. HiFi, Hi-C, Illumina, Illumina transcriptome sequencing and ONT transcriptome sequencing datasets are available under accession numbers SRR35229750^48^, SRR35229751^49^, SRR35229752^50^, SRR35229748^51^, and SRR35229749^52^, respectively (Table 1). The genome assembly is available on the NCBI under BioSample accession SAMN47123544, assembly GCA_052426335.1^53^, and BioProject accession PRJNA1229169. All genome assembly and annotation results are publicly available on the Figshare platform^54^.

## Technical Validation

In this study, we assessed the genome assembly quality using two complementary approaches. First, completeness was evaluated using BUSCO v5.7.1^26^ against the insecta_odb10 dataset (n = 1,367), yielding a completeness score of 98.4% with only 1.0% duplicated BUSCOs, indicating minimal redundancy. Second, to assess assembly accuracy, Illumina genomic reads, Illumina RNA-seq reads, ONT RNA-seq reads, and PacBio HiFi reads were mapped back to the assembly using Minimap2, and alignment rates were calculated using SAMtools. The mapping rates were 95.46%, 96.07%, 99.59%, and 99.46%, respectively, all exceeding 95%, demonstrating high assembly accuracy. Furthermore, the assembly exhibits a scaffold N50 of 27.479 Mb, a GC content of 39.10%, a chromosome-level scaffold anchoring rate of 96.91% across 13 pseudochromosomes, and QV values exceeding 60 for all chromosomes. Collectively, these metrics indicate that the *R. triangulispina* genome assembly achieves a high standard of quality in terms of continuity, completeness, and accuracy.

## Supplementary information

supplementary material

## Data Availability

The dataset described in this study is original and has been made publicly available for the first time. Raw sequencing reads and the assembled genome of *Rhopalopsole triangulispina* have been submitted to NCBI under BioProject accession number PRJNA1229169. Additionally, fully annotated data, including annotations of repetitive sequences, predicted gene models, and functional assignments, are available for download through Figshare at 10.6084/m9.figshare.30018145.v1.

## Code availability

In this study, no custom scripts were used for genome assembly and annotation. All bioinformatics software employed in data processing was applied according to the respective package documentation and standard operating procedures. Specific software versions, parameter settings, and corresponding references are provided in the manuscript.
