# Supplementary material for: Chromosome-level genome assembly of the stonefly Rhopalopsole triangulispina Mo and Li, 2025 (Plecoptera: Leuctridae)
Source: Sci Data. 2026 Jan 23;13:292. doi: 10.1038/s41597-026-06631-7 (PMC12929795; doi:10.1038/s41597-026-06631-7)
Supplement: Supplementary file 1 — supplementary material [file 41597_2026_6631_MOESM1_ESM.docx]

**Table S1. Repetitive sequence annotation statistics of *Rhopalopsole triangulispina*.**

| **Class** | **Copies** | **Length (bp)** | **Percentage of the genome** |
| --- | --- | --- | --- |
| **DNA** | 1116 | 243150 | 0.07% |
| Academ-1 | 65 | 8229 | 0.00% |
| Academ-2 | 6 | 333 | 0.00% |
| Academ-H | 3 | 164 | 0.00% |
| CMC | 1 | 70 | 0.00% |
| CMC-Chapaev | 43 | 2959 | 0.00% |
| CMC-Chapaev-3 | 18 | 1079 | 0.00% |
| CMC-EnSpm | 3147 | 475351 | 0.14% |
| CMC-Transib | 63 | 9287 | 0.00% |
| Crypton | 6 | 236 | 0.00% |
| Crypton-A | 9 | 770 | 0.00% |
| Crypton-C | 9 | 769 | 0.00% |
| Crypton-F | 28 | 4255 | 0.00% |
| Crypton-H | 38 | 3304 | 0.00% |
| Crypton-I | 1 | 38 | 0.00% |
| Crypton-S | 38 | 8126 | 0.00% |
| Crypton-V | 61 | 3727 | 0.00% |
| Dada | 93 | 20263 | 0.01% |
| Ginger-1 | 87 | 13758 | 0.00% |
| Ginger-2 | 29 | 3880 | 0.00% |
| IS3EU | 105 | 39196 | 0.01% |
| Kolobok | 16 | 2053 | 0.00% |
| Kolobok-E | 213 | 98525 | 0.03% |
| Kolobok-H | 1 | 92 | 0.00% |
| Kolobok-Hydra | 235 | 75021 | 0.02% |
| Kolobok-T2 | 272 | 83892 | 0.02% |
| MULE | 3 | 245 | 0.00% |
| MULE-F | 1 | 36 | 0.00% |
| MULE-MuDR | 3646 | 503998 | 0.15% |
| MULE-NOF | 352 | 203448 | 0.06% |
| MULE-Ricksha | 1 | 58 | 0.00% |
| Maverick | 1077 | 886834 | 0.26% |
| Maverick-Mavirus | 2 | 79 | 0.00% |
| Merlin | 195 | 56052 | 0.02% |
| Novosib | 41 | 9003 | 0.00% |
| P | 2226 | 823711 | 0.24% |
| P-Fungi | 5 | 265 | 0.00% |
| PIF | 1 | 72 | 0.00% |
| PIF-HarbS | 804 | 233003 | 0.07% |
| PIF-Harbinger | 2357 | 397758 | 0.11% |
| PIF-ISL2EU | 256 | 84468 | 0.02% |
| PIF-Spy | 32 | 1417 | 0.00% |
| PiggyBac | 185 | 22423 | 0.01% |
| PiggyBac-X | 10 | 558 | 0.00% |
| Sola-1 | 132 | 62738 | 0.02% |
| Sola-2 | 12219 | 2719849 | 0.78% |
| Sola-3 | 100 | 14813 | 0.00% |
| TcMar | 79 | 4850 | 0.00% |
| TcMar-Ant1 | 14 | 1139 | 0.00% |
| TcMar-Cweed | 5 | 1265 | 0.00% |
| TcMar-Fot1 | 1349 | 676420 | 0.19% |
| TcMar-IS885 | 1 | 89 | 0.00% |
| TcMar-ISRm11 | 39 | 7361 | 0.00% |
| TcMar-Mariner | 27447 | 5199110 | 1.50% |
| TcMar-Pogo | 29 | 3910 | 0.00% |
| TcMar-Sagan | 1 | 41 | 0.00% |
| TcMar-Stowaway | 20 | 2034 | 0.00% |
| TcMar-Tc1 | 24627 | 6520933 | 1.88% |
| TcMar-Tc2 | 21 | 2540 | 0.00% |
| TcMar-Tc4 | 35 | 1806 | 0.00% |
| TcMar-Tigger | 75 | 19594 | 0.01% |
| TcMar-m44 | 21 | 3049 | 0.00% |
| Zator | 11 | 652 | 0.00% |
| Zisupton | 199 | 37886 | 0.01% |
| hAT | 8652 | 757192 | 0.22% |
| hAT-Ac | 4541 | 465577 | 0.13% |
| hAT-Blackjack | 3429 | 681146 | 0.20% |
| hAT-Charlie | 6284 | 2648123 | 0.76% |
| hAT-Pegasus | 13 | 975 | 0.00% |
| hAT-Tag1 | 1688 | 334181 | 0.10% |
| hAT-Tip100 | 12733 | 1886539 | 0.54% |
| hAT-hAT1 | 2 | 122 | 0.00% |
| hAT-hAT19 | 8420 | 2078026 | 0.60% |
| hAT-hAT5 | 915 | 114184 | 0.03% |
| hAT-hAT6 | 2 | 142 | 0.00% |
| hAT-hATm | 1483 | 475212 | 0.14% |
| hAT-hATw | 15 | 6425 | 0.00% |
| hAT-hATx | 8 | 313 | 0.00% |
| hAT-hobo | 10 | 441 | 0.00% |
| **LINE** | 329 | 60025 | 0.02% |
| CR1 | 2245 | 549527 | 0.16% |
| CR1-Zenon | 67 | 6674 | 0.00% |
| CRE | 11 | 798 | 0.00% |
| CRE-Ambal | 28 | 12590 | 0.00% |
| CRE-Odin | 8 | 358 | 0.00% |
| Deceiver | 1 | 49 | 0.00% |
| Dong-R4 | 301 | 247240 | 0.07% |
| Dualen | 8 | 450 | 0.00% |
| I | 3076 | 1291755 | 0.37% |
| I-Jockey | 1225 | 541020 | 0.16% |
| L1 | 1287 | 156897 | 0.05% |
| L1-DRE | 9 | 337 | 0.00% |
| L1-Tx1 | 190 | 54596 | 0.02% |
| L2 | 9608 | 3552739 | 1.02% |
| Penelope | 2192 | 595517 | 0.17% |
| Proto1 | 5 | 326 | 0.00% |
| Proto2 | 10 | 643 | 0.00% |
| R1 | 526 | 335940 | 0.10% |
| R1-LOA | 28 | 1935 | 0.00% |
| R2 | 471 | 326215 | 0.09% |
| R2-Hero | 13 | 609 | 0.00% |
| R2-NeSL | 243 | 59327 | 0.02% |
| RTE | 13 | 612 | 0.00% |
| RTE-BovB | 25150 | 3246207 | 0.94% |
| RTE-ORTE | 3 | 147 | 0.00% |
| RTE-RTE | 25 | 1752 | 0.00% |
| RTE-X | 3739 | 1254974 | 0.36% |
| Rex-Babar | 67 | 13564 | 0.00% |
| Tad1 | 40 | 2180 | 0.00% |
| **LTR** | 172 | 13941 | 0.00% |
| Caulimovirus | 132 | 17920 | 0.01% |
| Copia | 2868 | 1297258 | 0.37% |
| DIRS | 200 | 65755 | 0.02% |
| DIRS-Q | 2 | 271 | 0.00% |
| ERV-Foamy | 1 | 90 | 0.00% |
| ERV-Lenti | 2 | 76 | 0.00% |
| ERV1 | 568 | 63817 | 0.02% |
| ERV4 | 12 | 759 | 0.00% |
| ERVK | 728 | 281033 | 0.08% |
| ERVL | 63 | 9868 | 0.00% |
| ERVL-MaLR | 10 | 554 | 0.00% |
| Gypsy | 20619 | 7997316 | 2.30% |
| Ngaro | 1028 | 294986 | 0.08% |
| Pao | 893 | 330143 | 0.10% |
| Unknown | 7684 | 2083888 | 0.60% |
| **Other** | 1 | 71 | 0.00% |
| DNA_virus | 2 | 90 | 0.00% |
| **PLE** | – | – | – |
| Chlamys | 648 | 191170 | 0.06% |
| Naiad | 15 | 708 | 0.00% |
| **RC** | – | – | – |
| Helitron | 16179 | 7394471 | 2.13% |
| Helitron-2 | 5 | 139 | 0.00% |
| Retroposon | 11 | 1801 | 0.00% |
| L1-dep | 182 | 18770 | 0.01% |
| L1-derived | 6 | 687 | 0.00% |
| L2-derived | 1 | 61 | 0.00% |
| RTE-derived | 5 | 214 | 0.00% |
| SVA | 1 | 133 | 0.00% |
| sno | 6 | 228 | 0.00% |
| **SINE** | 16 | 372 | 0.00% |
| 5S | 1 | 67 | 0.00% |
| 7SL | 1 | 289 | 0.00% |
| Alu | 9 | 317 | 0.00% |
| B2 | 4 | 243 | 0.00% |
| B4 | 115 | 68378 | 0.02% |
| Core | 1 | 114 | 0.00% |
| ID | 6851 | 843056 | 0.24% |
| MIR | 18 | 535 | 0.00% |
| U-L1 | 9 | 858 | 0.00% |
| tRNA | 103793 | 16860008 | 4.86% |
| tRNA-5S | 2 | 147 | 0.00% |
| tRNA-7SL | 1 | 5 | 0.00% |
| tRNA-CR1 | 2 | 110 | 0.00% |
| tRNA-Core | 21 | 931 | 0.00% |
| tRNA-Core-RTE | 17 | 432 | 0.00% |
| tRNA-Deu | 7133 | 593250 | 0.17% |
| tRNA-Deu-L2 | 12 | 571 | 0.00% |
| tRNA-Deu-RTE | 2 | 62 | 0.00% |
| tRNA-I | 5 | 218 | 0.00% |
| tRNA-L2 | 12 | 454 | 0.00% |
| tRNA-Meta | 7656 | 892515 | 0.26% |
| tRNA-RTE | 176 | 23852 | 0.01% |
| tRNA-V | 6 | 331 | 0.00% |
| tRNA-V-CR1 | 2 | 57 | 0.00% |
| tRNA-V-L2 | 1 | 57 | 0.00% |
| **Unknown** | 334639 | 72580849 | 20.91% |
| TATE | 1 | 60 | 0.00% |
| centromeric | 33 | 3163 | 0.00% |
| **Total_interspersed** | 695017 | 153303154 | 44.16% |
| **Low_complexity** | 8275 | 399432 | 0.12% |
| **Satellite** | 4420 | 677740 | 0.20% |
| acro | 4 | 326 | 0.00% |
| centr | 15 | 2638 | 0.00% |
| macro | 2 | 39 | 0.00% |
| subtelo | 5 | 391 | 0.00% |
| **Simple_repeat** | 63893 | 12588910 | 3.63% |
| **rRNA** | 854 | 1356628 | 0.39% |
| **snRNA** | 31 | 3697 | 0.00% |
| **tRNA** | 302 | 17098 | 0.00% |
| **Total** | 772818 | 168350053 | 48.50% |

**Table S2. Non-coding RNA annotation statistics of *Rhopalopsole triangulispina*.**

| **Class** | **Copies** | **Description** | **Comment** |
| --- | --- | --- | --- |
| **ribozyme** |  |  |  |
| RNaseP_nuc | 1 | ribozyme | Nuclear RNase P |
| **miRNA** |  |  |  |
| bantam | 1 | miRNA | microRNA bantam |
| let-7 | 1 | miRNA | let-7 microRNA precursor |
| mir-1 | 3 | miRNA | mir-1 microRNA precursor family |
| mir-10 | 2 | miRNA | mir-10 microRNA precursor family |
| mir-1000 | 1 | miRNA | mir-1000 microRNA precursor family |
| mir-11 | 1 | miRNA | mir-11 microRNA precursor family |
| mir-1175 | 1 | miRNA | mir-1175 microRNA precursor family |
| mir-124 | 1 | miRNA | mir-124 microRNA precursor family |
| mir-133 | 1 | miRNA | mir-133 microRNA precursor family |
| mir-137 | 1 | miRNA | microRNA mir-137 |
| mir-14 | 1 | miRNA | microRNA mir-14 |
| mir-184 | 1 | miRNA | microRNA mir-184 |
| mir-190 | 1 | miRNA | microRNA mir-190 |
| mir-2 | 4 | miRNA | mir-2 microRNA precursor |
| mir-210 | 1 | miRNA | microRNA mir-210 |
| mir-219 | 1 | miRNA | mir-219 microRNA precursor family |
| mir-252 | 2 | miRNA | microRNA mir-252 |
| mir-263 | 2 | miRNA | mir-263 microRNA precursor family |
| mir-276 | 1 | miRNA | microRNA mir-276 |
| mir-2765 | 1 | miRNA | mir-2765 microRNA precursor family |
| mir-277 | 1 | miRNA | microRNA mir-277 |
| mir-278 | 1 | miRNA | microRNA mir-278 |
| mir-2796 | 1 | miRNA | mir-2796 microRNA precursor family |
| mir-282 | 1 | miRNA | microRNA mir-282 |
| mir-29 | 1 | miRNA | mir-29 microRNA precursor |
| mir-305 | 1 | miRNA | microRNA mir-305 |
| mir-31 | 1 | miRNA | microRNA mir-31 |
| mir-315 | 1 | miRNA | microRNA mir-315 |
| mir-317 | 1 | miRNA | microRNA mir-317 |
| MIR397 | 1 | miRNA | microRNA MIR397 |
| mir-449 | 1 | miRNA | microRNA mir-449 |
| mir-46 | 1 | miRNA | mir-46/mir-47/mir-281 microRNA precursor family |
| mir-598 | 3 | miRNA | microRNA mir-598 |
| mir-67 | 1 | miRNA | microRNA mir-67 |
| mir-7 | 1 | miRNA | mir-7 microRNA precursor |
| mir-71 | 1 | miRNA | microRNA mir-71 |
| mir-750 | 1 | miRNA | mir-750 microRNA precursor family |
| mir-8 | 1 | miRNA | mir-8/mir-141/mir-200 microRNA precursor family |
| MIR811 | 1 | miRNA | microRNA MIR811 |
| mir-9 | 3 | miRNA | mir-9/mir-79 microRNA precursor family |
| mir-927 | 1 | miRNA | microRNA mir-927 |
| mir-929 | 1 | miRNA | microRNA mir-929 |
| mir-932 | 1 | miRNA | microRNA mir-932 |
| mir-965 | 2 | miRNA | mir-965 microRNA precursor family |
| mir-971 | 1 | miRNA | mir-971 microRNA precursor family |
| mir-981 | 1 | miRNA | microRNA mir-981 |
| mir-iab-4 | 1 | miRNA | mir-iab-4 microRNA precursor family |
| **lncRNA** |  |  |  |
| Sphinx_1 | 1 | lncRNA | Sphinx_ conserved region 1 |
| Sphinx_2 | 1 | lncRNA | Sphinx conserved region 2 |
| **rRNA** |  |  |  |
| 5_8S_rRNA | 115 | rRNA | 5.8S ribosomal RNA |
| 5S_rRNA | 707 | rRNA | 5S ribosomal RNA |
| LSU_rRNA_archaea | 4 | rRNA | Archaeal large subunit ribosomal RNA |
| LSU_rRNA_eukarya | 158 | rRNA | Eukaryotic large subunit ribosomal RNA |
| SSU_rRNA_archaea | 1 | rRNA | Archaeal small subunit ribosomal RNA |
| SSU_rRNA_bacteria | 1 | rRNA | Bacterial small subunit ribosomal RNA |
| SSU_rRNA_eukarya | 163 | rRNA | Eukaryotic small subunit ribosomal RNA |
| SSU_rRNA_microsporidia | 4 | rRNA | Microsporidia small subunit ribosomal RNA |
| **snRNA** |  |  |  |
| SCARNA8 | 4 | snRNA; snoRNA; scaRNA | Small Cajal body specific RNA 8 |
| snoMe28S-G3255 | 1 | snRNA; snoRNA; CD-box | Small nucleolar RNA Me28S-Gm3255 |
| snoR38 | 1 | snRNA; snoRNA; CD-box | Small nucleolar RNA R38 |
| SNORA16 | 2 | snRNA; snoRNA; HACA-box | Small nucleolar RNA SNORA16B/SNORA16A family |
| SNORA53 | 1 | snRNA; snoRNA; HACA-box | Small nucleolar RNA SNORA53 |
| SNORD18 | 1 | snRNA; snoRNA; CD-box | Small nucleolar RNA SNORD18 |
| SNORD24 | 1 | snRNA; snoRNA; CD-box | Small nucleolar RNA SNORD24 |
| SNORD31 | 2 | snRNA; snoRNA; CD-box | Small nucleolar RNA SNORD31 |
| SNORD36 | 3 | snRNA; snoRNA; CD-box | Small nucleolar RNA SNORD36 |
| snosnR60_Z15 | 5 | snRNA; snoRNA; CD-box | Small nucleolar RNA snR60/Z15/Z230/Z193/J17 |
| snoU6-53 | 1 | snRNA; snoRNA; CD-box | Small nucleolar RNA U6-53/MBII-28 |
| snoU85 | 1 | snRNA; snoRNA; HACA-box | Small nucleolar RNA U85 |
| U1 | 6 | snRNA; splicing | U1 spliceosomal RNA |
| U11 | 1 | snRNA; splicing | U11 spliceosomal RNA |
| U2 | 11 | snRNA; splicing | U2 spliceosomal RNA |
| U3 | 6 | snRNA; snoRNA; CD-box | Small nucleolar RNA U3 |
| U4 | 6 | snRNA; splicing | U4 spliceosomal RNA |
| U4atac | 1 | snRNA; splicing | U4atac minor spliceosomal RNA |
| U5 | 6 | snRNA; splicing | U5 spliceosomal RNA |
| U6 | 8 | snRNA; splicing | U6 spliceosomal RNA |
| U6atac | 1 | snRNA; splicing | U6atac minor spliceosomal RNA |
| **tRNA** |  |  |  |
| tRNA-Ala | 20 | tRNA |  |
| tRNA-Arg | 26 | tRNA |  |
| tRNA-Asn | 9 | tRNA |  |
| tRNA-Asp | 18 | tRNA |  |
| tRNA-Cys | 4 | tRNA |  |
| tRNA-Gln | 10 | tRNA |  |
| tRNA-Glu | 14 | tRNA |  |
| tRNA-Gly | 21 | tRNA |  |
| tRNA-His | 6 | tRNA |  |
| tRNA-Ile | 14 | tRNA |  |
| tRNA-iMet/Met | 17 | tRNA |  |
| tRNA-Leu | 19 | tRNA |  |
| tRNA-Lys | 22 | tRNA |  |
| tRNA-Phe | 8 | tRNA |  |
| tRNA-Pro | 18 | tRNA |  |
| tRNA-SeC | 1 | tRNA |  |
| tRNA-Ser | 16 | tRNA |  |
| tRNA-Thr | 22 | tRNA |  |
| tRNA-Trp | 6 | tRNA |  |
| tRNA-Tyr | 7 | tRNA |  |
| tRNA-Val | 17 | tRNA |  |
| **Others** |  |  |  |
| Arthropod_7SK | 1 | Arthropod 7SK RNA |  |
| Histone3 | 825 | Histone 3’ UTR stem-loop |  |
| K_chan_RES | 4 | Potassium channel RNA editing signal |  |
| Metazoa_SRP | 1 | Metazoan signal recognition particle RNA |  |
| **Total** | 2411 |  |  |
